# Neurogranin: A Potential Biomarker of Neurological and Mental Diseases

**DOI:** 10.3389/fnagi.2020.584743

**Published:** 2020-10-06

**Authors:** Yang Xiang, Jiayan Xin, Weidong Le, Yongjian Yang

**Affiliations:** ^1^Institute of Neuroscience, Sichuan Academy of Medical Sciences and Sichuan Provincial People’s Hospital, School of Clinical Medicine, University of Electronic Science and Technology of China, Chengdu, China; ^2^Department of Neurology, General Hospital of Western Theater Command, Chengdu, China; ^3^North Sichuan Medical College, Nanchong, China; ^4^Department of Cardiovasology, General Hospital of Western Theater Command, Chengdu, China

**Keywords:** neurodegenerative disorder, mental disorder, biomarker, cerebrospinal fluid, neurogranin

## Abstract

Neurogranin (Ng) is a small protein usually expressed in granule-like structures in pyramidal cells of the hippocampus and cortex. However, its clinical value is not fully clear so far. Currently, Ng is proved to be involved in synaptic plasticity, synaptic regeneration, and long-term potentiation mediated by the calcium- and calmodulin-signaling pathways. Due to both the synaptic integrity and function as the growing concerns in the pathogenesis of a wide variety of neurological and mental diseases, a series of researches published focused on the associations between Ng and these kinds of diseases in the past decade. Therefore, in this review, we highlight several diseases, which include, but are not limited to, Alzheimer’s disease, Parkinson disease, Creutzfeldt–Jakob disease, neuro-HIV, neurosyphilis, schizophrenia, depression, traumatic brain injury, and acute ischemic stroke, and summarize the associations between cerebrospinal fluid or blood-derived Ng with these diseases. We propose that Ng is a potential and promising biomarker to improve the diagnosis, prognosis, and severity evaluation of these diseases in the future.

## Introduction

Neurogranin (Ng, also called RC3, p17, and BICKS) is a protein with a molecular weight of 7.5 kD and composed of 78 amino acids (Watson et al., 1990). It is often found in granule-like structures in pyramidal cells of the hippocampus and cortex, which gives rise to its name of “neurogranin” (Represa et al., 1990). Ng was discovered in 1990; however, its clinical value is not fully clear so far.

**FIGURE 1:**
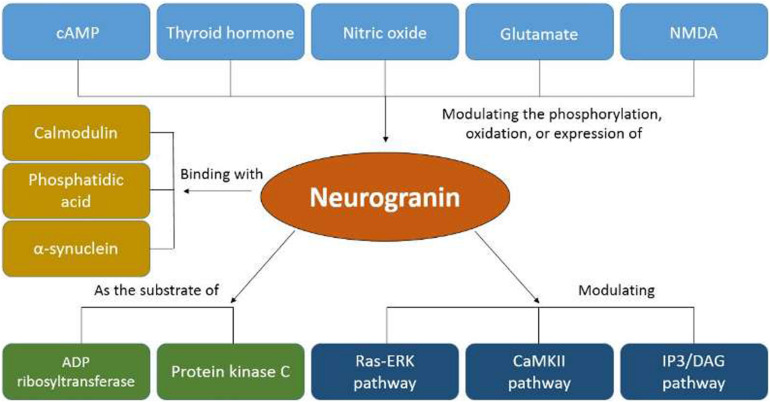
Cellular and regional distribution of neurogranin in the adult rat brain.

The mammalian Ng gene NRGN spans around 12.5 kbp and contains four exons and three introns (Martinez de Arrieta et al., 1997). The human Ng sequence predicts five amino acids encoded by exon 1 and 73 amino acids encoded by exon 2 (Martinez de Arrieta et al., 1997). However, the other two exons contain untranslated sequences (Martinez de Arrieta et al., 1997). The coding sequence homology of NRGN between humans and rats is 90% at the nucleic acid level and 96% at the protein level (Martinez de Arrieta et al., 1997). In early studies, Ng was found principally as a neuronal postsynaptic protein in the telencephalon of the adult rat, specifically located in the cell bodies and dendrites of neurons in the cerebral cortex, hippocampus, and striatum (Represa et al., 1990) (Figure 1). Thereafter, Ng was detected successively in the lung, spleen, and bone marrow with a low expression level (Diez-Guerra, 2010). Moreover, high and moderate levels of Ng were found in platelets and B type lymphocytes, respectively (Glynne et al., 2000; Gnatenko et al., 2003). A recent study first identified Ng expression in both human and mouse endothelia (Cheriyan et al., 2020).

**FIGURE 2:**
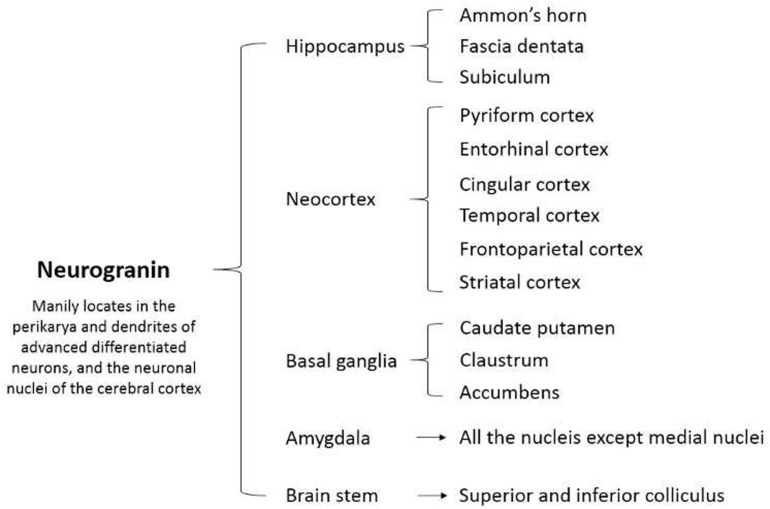
A diagram of the molecular and signaling pathways involved in neurogranin. cAMP, cyclic adenosine monophosphate; NMDA, *N*-methyl-D-aspartate; ADP, adenosine diphosphate; ERK, extracellular signal-regulated kinase; CaMK II, calmodulin-dependent protein kinase II; IP3, inositol 1,4,5-trisphosphate; DAG, diacylglycerol.

The present data strongly point that Ng is involved in the plasticity and regeneration of synapse mediated by the calcium- and calmodulin-signaling pathways. For instance, Zhong et al. (2009) found that Ng enhances the postsynaptic sensitivity an elevates the synaptic strength in an activity- and NMDAR- dependent manner (Zhong et al., 2009). Besides, the potentiation of synaptic transmission modulated by Ng mimics and occludes the long-term potentiation (Zhong et al., 2009). A recent study revealed that long-term blockade of NMDAR significantly decreases Ng expression (Garrido-Garcia et al., 2019). Moreover, the long-term bicuculline administration facilitates synaptic activity and increases Ng expression (Garrido-Garcia et al., 2019). Lentiviral expression of Ng results in the elevated density of both excitatory and inhibitory synapses (Garrido-Garcia et al., 2019). In addition, Ng is involved in a variety of biochemical processes and molecular interactions (Figure 2).

Of note, synaptic integrity and function are both the growing concerns in the pathogenesis of a wide variety of neurological and mental diseases (Fyfe, 2015; Hellwig et al., 2015; Yang et al., 2015; Zetterberg and Blennow, 2015; Bereczki et al., 2017; De Vos et al., 2017; Guha et al., 2018; Blennow et al., 2019). In animal experiments, it showed that mice lacking NRGN show a remarkable decline in hippocampus-dependent spatial memory and deficits in hippocampal long-term potentiation (Pak et al., 2000). Aging is associated with the cognitive decline as well as the decreased Ng levels in pyramidal neurons (Mons et al., 2001). Ng reduction and cognitive deficit detected in 5XFAD mice are restored after the intra-hippocampal injection with an Ng-expressing lentiviral vector (Jeon et al., 2018). On the basis of these basic studies, a series of researches published in the past decade focused on the associations between Ng and these kinds of diseases, which include, but are not limited to, AD, PD, CJD, HIV, infection (neuro-HIV), NS, TBI, AIS, and schizophrenia.

Therefore, in this review, we highlight and summarize several neurological and mental diseases associated with Ng and propose that Ng is a potential and promising biomarker to improve the diagnosis, prognosis, and severity evaluation of these diseases in the future.

## Associations Between Neurogranin and Neurological and Mental Diseases

Using the keywords, including “neurogranin,” “Ng,” “RC3,” and “BICKS,” we searched the clinical research articles involved in Ng via PUBMED and categorized them as per the kind of disease. An overview of the major clinical researches involved in Ng was currently published (Table 1).

**TABLE 1:** An overview of the major clinical researches involved in Ng published currently.

	Participants	Measures	Outcomes	Correlations	Study design	References
AD	ADD (*n* = 39) MCI-AD (*n* = 13) MCI-o (*n* = 29) Non-ADD (*n* = 14)	CSF Ng	MCI-AD ↑ vs. MCI-o ADD ↑ vs. MCI-o	Positively correlated with CSF tau and p-tau.	Case–control study	Hellwig et al., 2015; Kvartsberg et al., 2015b
	AD (*n* = 65) MCI (*n* = 61) CTRL (*n* = 37)	CSF Ng	Baseline CSF levels of Ng: AD ↑ vs. CTRL MCI-AD ↑ vs. sMCI Predicting progression from MCI to AD	Positively correlated with CSF T-tau and P-181 tau, but not with Aβ42.	Longitudinal study	Kester et al., 2015; Zetterberg and Blennow, 2015
	ADD (*n* = 95) MCI (*n* = 173) CTRL (*n* = 110)	CSF Ng	ADD ↑ vs. CTRL MCI ↑ vs. CTRL sMCI ↑ vs. CTRL pMCI ↑ vs. CTRL pMCI ↑ vs. sMCI	High baseline CSF Ng predicting cognitive decline as reflected by decreased MMSE.	Case–control study	Portelius et al., 2015
	ADD (*n* = 397) MCI (*n* = 114) FTD (*n* = 96) PDD (*n* = 29) DLB (*n* = 33) CTRL (*n* = 75)	CSF Ng and autopsy for the neuropathology	ADD ↑ vs. CTRL ADD ↑ vs. MCI AD biomarker-positive CTRL subjects ↑ vs. AD biomarker negative CTRL group ADD vs. PD ↓/PD MCI ↓/PDD ↓ ADD ↑ vs. FTD/ALS	Positively associated with: Aβ neuritic plaque and tau tangle pathology scores.	Prospective study	Portelius et al., 2018
	ADD (*n* = 100) MCI (*n* = 40) CTRL (*n* = 80)	CSF Ng	Both AD and MCI-AD: markedly decrease; The highest level in ADD; High CSF Ng levels at the MCI stage; Predicting progression to ADD.	Positively correlated with t-tau and p-tau, but no correlations with Aβ 1-42.	Case–control study	Kvartsberg et al., 2015a
	AD (*n* = 95) CTRL (*n* = 207)	CSF Ng	The mean (SE) AUC was 0.73 (0.04) for Ng to differentiate patients with early symptomatic AD from CTRL; Predicting future cognitive impairment (adjusted hazard ratio, 1.89).	CSF Ng level correlates with a whole brain and regional atrophy in AD, and the amyloid load in preclinical AD.	Cross-sectional and longitudinal observational study	Tarawneh et al., 2016
	AD (*n* = 10) MCI (*n* = 20) MCI-AD (*n* = 20) CTRL (*n* = 10)	Plasma NDEs levels of: PT-181-tau PS-396-tau Aβ 1-42 Ng	AD/MCI vs. CTRL: Plasma NDE levels of PT-181-tau PS-396-tau, and Abeta 1-42 ↑ Plasma NDE levels of Ng ↓	N/A	Case–control study	Winston et al., 2016
	Discovery stage: AD (*n* = 28) aMCI (*n* = 25) CTRL (*n* = 29) Validation stage: AD (*n* = 73) aMCI (*n* = 71) CTRL (*n* = 72) pre-AD (*n* = 160) CTRL (*n* = 160)	Blood nero-exosomal: GAP43 Ng SNAP25 Synaptotagmin 1	Discovery stage: AD ↓ vs. CTRL aMCI ↓ vs. CTRL aMCI ↑ vs. AD Validation stage: same with discovery stage The combination of exosomal biomarkers detected AD 5 to 7 years before cognitive impairment (AUC = 0.87–0.89)	Exosomal biomarker levels were correlated with those in CSF (*R*^2^ = 0.54–0.70).	Longitudinal and retrospectively study	Antonell et al., 2020; Jia et al., 2020
	MCI or ADD (in total *n* = 59) CTRL (*n* = 29)	Paired CSF/plasma samples	CSF: MCI ↑ vs. CTRL AD ↑ vs. CTRL plasma: AD vs. CTRL: no change	Positively correlated with CSF tau; Negatively correlated with CSFAβ 1-42/Aβ 1-40; No correlation between CSF and plasma Ng.	Case–control study	De Vos et al., 2015; Kvartsberg et al., 2015a
PD	PD (*n* = 52) PD Drug naïve (*n* = 30) CTRL (*n* = 87)	CSF Ng: Motor disease stage (Hoehn and Yahr scale) Cognitive performance (MoCA).	PD ↑ vs. CTRL	Associated with reduced cognition and higher motor disease stage.	Case–control study	Bereczki et al., 2017
	PD (*n* = 30) CTRL (*n* = 26)	CSF Aβ; a-synuclein; Ng; Cortical glucose metabolism.	PD ↓ vs. CTRL	Lower CSF Ng concentrations were found with more severe reductions on FDG-PET.	Longitudinal study	Selnes et al., 2017
	CTRL (*n* = 47) PD (*n* = 157) PDD (*n* = 29) AD (*n* = 124) MSA (*n* = 26)	CSF Ng	PD ↓/PDD ↓/ MSA ↓ /PSP ↓ vs. CTRL PD ↓/PDD ↓/ MSA ↓ /PSP ↓ vs. AD	No significant associations between Ng and clinical progression.	Case–control study	Hall et al., 2020
HD	CTRL (*n* = 12) HD gene expansion carriers (*n* = 20)	CSF Ng	HD vs. CTRL: No change	N/A	Case–control study	Byrne et al., 2018
CJD	AD (*n* = 46) CJD (*n* = 81) CTRL (*n* = 64)	CSF Ng T-tau NfL 14-3-3 protein postmortem brain tissue	CSF: CJD ↑ (4.75 times of CTRL) vs. CTRL AD ↑ (1.94 times of CTRL) vs. CTRL Differentiating CJD from AD (AUC = 0.85) Brain tissue: Ng reduced in AD, and more significantly in CJD	Showed a good correlation with tau, but did not correlate with NfL.	Case–control study	Blennow et al., 2019
	CJD (*n* = 38) Pre-AD (*n* = 21) MCI-AD (*n* = 56) ADD (*n* = 108) FTD (*n* = 34) CTR (*n* = 50)	CSF Ng	FTD ↓<CTRL < ADD ↑ <CJD ↑ Applying the AT(N) system, 62% of subjects were positive for neurodegeneration if Ng was used.	N/A	Unicentric cohort study	Antonell et al., 2020; Jia et al., 2020
Neuro-HIV	HIV-1-positive (*n* = 8) HIV-1-negative (*n* = 4)	The expression of Ng in FC tissues	HIV-1-positive ↓ vs. HIV-1-negative	Associated with a decreased level of CaMKII	Case-control study	Guha et al., 2018
	HIV-infected (*n* = 138) CTRL (*n* = 13)	CSF Ng	HIV-infected individuals vs. CTRL: No change	N/A	Cross-sectional study	Sinharay and Hammoud, 2019; Yilmaz et al., 2019
Neurosyphilis	NS (*n* = 13) GPI (*n* = 55) AD (*n* = 23)	CSF/plasma: Ng, Aβ, BACE1	CSF Ng, BACE1, and tau, as well as plasma BACE1 levels, were significantly different among groups.	CSF tau and plasma Ng correlated with cognitive scale scores	Case–control study	Zhang et al., 2020
Schizophrenia	Schizophrenia (*n* = 7) CTRL (*n* = 7)	Prefrontal cortex expression of Ng in pyramidal cells in layers III and V in area 9 and 32.	A marked decrease in Ng immunostaining in both areas 9 and 32 of the prefrontal cortex.	N/A	Case–control study	Broadbelt et al., 2006
Depression	Major depression (*n* = 12)	Whether the ECT will change CSF Ng	CSF Ng concentrations do not change before and after a course of ECT	Baseline Ng levels were positively correlated with the therapeutic response.	Prospective study	Kranaster et al., 2017
FEP	FEP patients (*n* = 40) CTRL (*n* = 20)	CSF Ng	FEP patients ↓ vs. CTRL	N/A	Longitudinal study	Santillo et al., 2019
TBI	TBI patients (*n* = 76) CTRL (*n* = 150)	Serum Ng	TBI patients ↑ vs. CTRL with an ROC for diagnosing TBI of 0.72	N/A	Case–control study	Yang et al., 2015
	CTRL (*n* = 328) mTBI (*n* = 179)	Serum Ng	mTBI patients ↑ vs. CTRL	N/A	Prospective observational study	Peacock et al., 2017
AIS	AIS (*n* = 50)	Paired CSF/plasma Ng	Ng was elevated in both CSF and plasma.	Positively correlated with infarct volume	Prospective study	De Vos et al., 2017

*MCI-AD, MCI due to AD; MCI-o, MCI not due to AD; aMCI, amnestic MCI; CTRL, control; sMCI, stable MCI; pMCI, progressive MCI; Ng, neurogranin; AD, Alzheimer’s disease; PD, Parkinson disease; CJD, Creutzfeldt–Jakob disease; TBI, traumatic brain injury; mTBI, mild TBI; AIS, acute ischemic stroke; CSF, cerebrospinal fluid; Aβ, amyloid β; t-tau, total tau; p-tau, phosphorylated tau; MCI, mild cognitive impairment; HD, Huntington disease; FTD, frontotemporal dementia; DLB, dementia with Lewy bodies; PDD, PD with dementia; NfL, neurofilament light; neuro-HIV, neuro-human immunodeficiency virus; FC, frontal cortex; MMSE, Mini–Mental State Examination; ECT, electroconvulsive therapy; MoCA, the Montreal Cognitive Assessment scores; FEP, first episode psychosis; ROC, receiver operating characteristic; NDEs, neuronal-derived exosomes; NS, neurosyphilis; GPI, general paresis of the insane; BACE1, precursor protein cleaving enzyme; N/A, not applicable due to lack of evidence; ↑, increased level; ↓, decreased level.*

### Cerebrospinal Fluid Neurogranin in Neurodegenerative Disorders

Neurodegenerative disorders (NDs) are characterized by progressive dysfunction of neurons, glias, synapses, as well as the neural networks (Kovacs, 2016, 2017). A critical feature of NDs is the aggregation and deposition of variants of physiological proteins in the CNS (Kovacs, 2016, 2017). Both neurons and glias have the capacity to accumulate these pathological variants (Kovacs, 2016, 2017). NDs can be broadly classified by their clinical presentations, most of which are the disorders of movement, cognition, mentation, or behavior. A small portion of patients develop pure syndromes, but most patients show mixed clinical features (Dugger and Dickson, 2017). AD and PD are the two kinds of the most common NDs. The occurrence of these types of NDs is usually in middle or old age, and the incidence is elevated with an increasing life expectancy of the population. In general, the diagnostic gold criteria of diverse NDs are neuropathological evaluation at autopsy. In comparison, the detectable biomarkers *in vivo* are supposed to improve the diagnosis, stratification, and prognosis of patients (Kovacs, 2016).

#### Cerebrospinal Fluid/Plasma Neurogranin in Alzheimer’s Disease

As the most common kind of dementing disease, AD is a relentlessly progressive and fatal disorder of CNS, which begins approximately 10–15 years before the clinical manifestations (Rafii, 2016). Pathologically, AD is characterized by both certain hallmarks in the brain, including the extracellular plaques composed of Aβ peptide and the intracellular neurofibrillary tangles composed of the hyperphosphorylated tau protein (Blennow et al., 2006). Undoubtedly, the core CSF biomarkers of Aβ reflecting brain amyloidosis, t-tau reflecting neurodegeneration intensity, and p-tau that is related to tau pathology, have good diagnostic accuracy in clinical practice. However, in view of the multifactorial pathogenesis of AD and the overlapping pathology with other kinds of dementia, it is necessary to integrate the core CSF biomarkers with other novel biomarkers that are capable of reflecting different aspects of neuropathology. Synaptic degeneration is an essential component of AD pathophysiology, which is present in early disease stages (Masliah et al., 2001; Scheff et al., 2007). An increasing amount of data suggests that synaptic dysfunction is associated with cognitive decline and ahead of neuronal degeneration (DeKosky and Scheff, 1990). Thus, the biomarkers reflecting the integrity and plasticity of synapses may be useful for the early diagnosis and prognosis of AD.

Many of clinical studies support the findings that the levels of CSF Ng are higher in AD or MCI patients than those in healthy controls (HCs) or non-AD dementia patients (Fyfe, 2015; Hellwig et al., 2015; Kester et al., 2015; Portelius et al., 2015). Higher levels of CSF Ng are positively correlated to higher scores of Aβ neuritic plaques and tau tangles pathology (Portelius et al., 2018). High levels of CSF Ng in AD and prodromal AD have been verified in several subsequent studies (Hellwig et al., 2015; Kvartsberg et al., 2015a).

A study explored the correlations between baseline CSF Ng levels with baseline and longitudinal cognitive decline, brain atrophy, and glucose metabolism (Portelius et al., 2015). They found that high baseline levels of CSF Ng in the MCI patients are associated with the longitudinal decline of hippocampal volume and cortical glucose metabolism at clinical follow-up (Portelius et al., 2015). Further, within the progressive MCI group, elevated CSF Ng levels correlate with accelerated deterioration in Alzheimer’s disease Assessment Scale—cognitive subscale (Portelius et al., 2015). In a recent meta-analysis, it revealed that the CSF Ng level is significantly higher in MCI patients progressed to AD than that in stable MCI patients (Mavroudis et al., 2019).

A cross-sectional and longitudinal observational study of cognitive decline between the symptomatic AD patients and cognitively normal controls proved that the CSF levels of Ng can develop the diagnosis and prognosis for early symptomatic AD that is comparable with other CSF biomarkers of AD (Tarawneh et al., 2016). Importantly, CSF Ng enhances the comprehensive capacity of these biomarkers to predict future cognitive decline in the cognitively normal controls (Tarawneh et al., 2016).

Additionally, the data of Ng expression in postmortem brain tissues of AD demonstrated that the elevated CSF Ng levels are in accordance with the decreased Ng levels in the cerebral cortex and hippocampus (Blennow et al., 2019). Ng levels in brain tissues of AD do not differ between early and late Braak stages, indicating that synaptic loss is not only a late-stage pathological feature (Blennow et al., 2019). Therefore, CSF Ng is a promising biomarker for early diagnosis and progression prediction of AD, which could be a useful complement to the panel of AD biomarkers currently.

For clinical applications, sample collection needs to be as accessible and reproducible as possible. However, no significant differences were found in plasma levels of Ng between AD patients and controls (De Vos et al., 2015), indicating the necessity of developing other kinds of Ng-related biomarkers from the blood. In that regard, a pilot study investigated the blood-derived Ng and revealed that the concentrations of Ng in the plasmatic NDEs are significantly lower in AD compared with the controls and correlate with the progression from MCI to AD (Winston et al., 2016). A recent meta-analysis uncovered that compared with the cognitively normal controls, the levels of plasmatic NDEs Ng in AD patients have an obvious decrease (Liu et al., 2020). Moreover, a recent study confirmed the difference in plasmatic NDEs Ng between AD patients and controls (Jia et al., 2020). Furthermore, the plasmatic exosomal levels of Ng are found to be correlated with CSF Ng levels. Also, the plasmatic exosomal Ng distinguishes AD with amnestic MCI and controls with the highest accuracy among all the plasmatic exosomal synaptic protein candidates, including growth-associated protein 43, Ng, synaptosome-associated protein 25, and synaptotagmin 1 (Jia et al., 2020).

The NIA-AA Research Framework, published in 2018, emphasized the necessity of a biological definition of AD and established the A/T/(N) biomarker classification system (Jack et al., 2018). In the framework, “A,” “T” and “(N)” stand for Aβ, tau, and neurodegeneration, respectively. It is generally recognized that “N” includes the cellular injury, regional volume loss of the brain, and the destruction of system-level circuits (Jack et al., 2018). Taken together, as a postsynaptic protein, the current evidence suggests that Ng is a promising biomarker reflecting synaptic dysfunction in AD. The value of CSF Ng in the diagnosis and prediction of AD has been clarified, but the relationship between blood-derived Ng and AD still needs further study.

#### Cerebrospinal Fluid Neurogranin in Parkinson Disease

Characterized by the loss of nigrostriatal dopaminergic neurons, PD is the second most common primary ND of the CNS, whose major clinical manifestation is the development of movement disorder (Barker and Transeuro consortium, 2019). Synaptic dysfunction is an early change in PD, which has been shown in a previous animal study (Yarnall et al., 2013). It proved that the neurons expressing Ng in the cortex degenerate in the late stage of PD (Yarnall et al., 2013). Besides, the levels of phosphorylated Ng are also lower in the superior temporal cortex in PD patients (Koob et al., 2014).

A study enrolled 52 PD patients and 87 HCs, measured the CSF concentrations of Ng, and explored the associations between Ng with motor symptoms (evaluated by Hoehn and Yahr scale) as well as cognitive symptoms (evaluated by the Montreal Cognitive Assessment scores) (Bereczki et al., 2017). It showed significant associations between increased concentrations of CSF Ng and cognitive impairment in the PD group, and CSF Ng is increased in PD patients in a disease-specific manner and associated with the severity of cognitive decline and motor disorder (Bereczki et al., 2017). Confusingly, a subsequent study showed the inconsistent results that enrolled 30 patients with mild-to-moderate PD and 26 HCs and tested the correlation between hypometabolism, CSF Aβ, CSF Ng, and CSF α-synuclein (Selnes et al., 2017). It showed that the CSF Ng levels are significantly lower in mild-to-moderate PD than those in controls and associated with CSF Aβ levels, CSF α-synuclein levels, and motor stage (Selnes et al., 2017). A prospective study showed that the Ng levels are significantly lower in PD, PD with MCI, and PDD) relative to AD dementia (Portelius et al., 2018). A recent study tested the CSF Ng in patients with PD, PDD, AD, and HCs and investigated the possible correlations between CSF Ng with cognitive and motor impairment (Hall et al., 2020). They found that Ng is decreased in patients with PD and PDD compared with the HCs and AD patients, respectively (Hall et al., 2020). Nevertheless, they did not find that Ng correlates with a motor disorder, cognitive impairment, longitudinal cognitive decline, or the progression to dementia in PD (Hall et al., 2020).

To sum up, the research on PD and Ng is booming currently, but the diagnostic value of Ng in CSF still needs to be further explored. More importantly, the correlation between blood Ng and PD remains unclear.

#### Cerebrospinal Fluid Neurogranin in Huntington Disease

As an autosomal dominant inheritance disease, HD is devastating to patients and their families, which is caused by an expanded trinucleotide repeat of CAG in the gene of huntingtin (Bates et al., 2015). There is evidence that synaptic dysfunction is a critical feature in HD pathogenesis (Smith et al., 2005; Sepers and Raymond, 2014). The whole-brain gene expression study in postmortem HD patient brains proved that the NRGN is one of the most robustly downregulated genes in HD caudate compared with the controls (Hodges et al., 2006; Runne et al., 2007). However, Byrne et al. (2018) quantified Ng and triggering receptor expressed on myeloid cells-2 in CSF samples from HD mutation carriers and controls and found that CSF Ng levels do not significantly differ between HD and HCs (Byrne et al., 2018). In addition, it did not find the significant associations between CSF Ng levels and the disease burden score, total functional capacity, or motor score (Byrne et al., 2018).

In a word, Ng-related research in HD is still far behind that of AD and PD. This may attribute to the type and characteristics of the disease or the limitation of the current testing methods of Ng.

#### Cerebrospinal Fluid Neurogranin in Creutzfeldt–Jakob Disease

Creutzfeldt–Jakob disease is a rapidly progressive and fatal neurodegenerative disease that is caused by misfolded, transmissible proteinaceous infectious particles (Uttley et al., 2020). One fundamental characteristic of CJD is synaptic degeneration and disorganization, resulting in neuronal loss and spongiform changes (Blennow et al., 2019). Actually, over a 30% reduction of the certain synaptic index in the brain has been found in prion disease compared with the controls (Clinton et al., 1993).

Blennow et al. (2019) investigated CSF Ng, t-tau, neurofilament light, and 14-3-3 protein in CJD (*n* = 81), AD (*n* = 46), and neurological controls (NCs, *n* = 64). The accuracy of Ng that differentiates the three groups and Ng expression in postmortem brain tissue was evaluated. They found that CJD has the highest levels of CSF Ng, which is helpful in the prediction of prognosis of CJD, is not influenced by age or sex, and is dependent on disease subtype (Blennow et al., 2019). In detail, CSF Ng is elevated in MM1/MV1 molecular subtypes compared with the VV2 subtype, which is in line with the severity of cortical pathological affectation (Blennow et al., 2019). However, the authors did not consider CSF Ng as a specific marker of synaptic degeneration but rather a marker of neuronal damage (Blennow et al., 2019). A recent study had a unicentric cohort of 353 participants, including HC subjects, AD, frontotemporal dementia (FTD), and CJD (Antonell et al., 2020). They analyzed and compared the diagnostic accuracy and differentiating capacity of four noncore biomarkers, which stand for the distinct aspects of the neurodegeneration process (Antonell et al., 2020). The rank of CSF Ng concentrations from lower to higher is FTD < HC < AD < CJD, which is in concordance with previously published data. Comparing their capacity in differentiating among neurodegenerative dementias, CSF Ng shows the significant differences across all three groups (AD, FTD, and CJD) (Antonell et al., 2020).

At present, the relation of Ng and CJD is still scatteredly reported, in which CSF Ng has been evaluated for diagnosis and differential diagnosis, and the results are relatively consistent, suggesting that CSF Ng has the potential to be a CJD biomarker.

#### Cerebrospinal Fluid Neurogranin in Other Neurodegenerative Disorders

In addition to the AD, PD, and HD, some scattered studies about CSF Ng in other NDs, including FTD, DLB, progressive supranuclear palsy (PSP), and multiple system atrophy (MSA), have been published so far (Wellington et al., 2016; Portelius et al., 2018). An optimized immunoassay was introduced to analyze CSF Ng in a retrospective cohort, which showed FTD does not have significantly elevated CSF Ng concentrations compared with controls (Wellington et al., 2016). Of note, CSF Ng concentrations are slightly higher in speech variant frontotemporal dementia compared with behavioral variant frontotemporal dementia (Wellington et al., 2016). A study investigated CSF levels of Ng and other two synaptic proteins in FTD. CSF samples were analyzed in 66 patients in the FTD spectrum and 19 HCs. Patients were stratified as per their tau-to-Aβ42 ratio (tau/Aβ42) (Clarke et al., 2019). In detail, patients with a ratio of >1 were considered as undergoing the likely AD pathology (“AD biomarker” group [*n* = 18]), and patients with a ratio <1 were considered as undergoing the likely FTD pathology (“FTD biomarker” group [*n* = 48]) (Clarke et al., 2019). However, no CSF synaptic proteins showed a pathological abnormality in the “FTD biomarker” group, and the higher CSF concentrations of Ng appear to be more related to AD pathology (Clarke et al., 2019).

In a study in which a total of 129 postmortem human brain samples were analyzed in brain regional-specific manner, it found that Ng levels are reduced across the brain regions in all the three dementia groups (DLB, PDD, and AD) compared with the controls (Bereczki et al., 2016). The most significant changes reflecting synaptic dysfunction were found in DLB patients, followed by patients with PDD and AD (Bereczki et al., 2016). The authors suggested that the proposition that synaptic biomarkers predicting cognitive decline in AD is supposed to be extended to DLB (Bereczki et al., 2016). In contrast, another retrospective cohort study did not show the significant differences in CSF Ng concentrations between DLB and controls (Wellington et al., 2016).

### Cerebrospinal Fluid Neurogranin in Infectious Diseases of the Central Nervous System

Infectious diseases of the CNS have a sizable effect on local health-care systems and economies (Vora et al., 2014). The change in mental status induced by the inflammation is a hallmark of neurotropic pathogen infections of the CNS (Klein et al., 2017). Pathogens, including bacteria, viruses, fungi, and parasites, can invade the brain parenchyma and give rise to the inflammation and/or the infection of both meningeal and parenchymal compartments, which lead to the dysfunction of neurons, glia cells, and the neural networks (Cain et al., 2019). On the basis of the published studies so far, we mainly summarize the relationship between Ng and the following two infectious diseases of the nervous system.

#### Cerebrospinal Fluid Neurogranin in Neuro-Human Immunodeficiency Virus Infection

Soon after transmission, HIV can be detected in the CSF in most patients (Valcour et al., 2012). The antiretroviral therapy has decreased the rates of mortality and morbidity in HIV-positive (HIV^+^) patients and has decreased the incidence of HIV-associated dementia, which is the most severe stage of neuro-HIV (Sinharay and Hammoud, 2019). Synaptic disruption is crucial in the mechanisms of cognitive impairment in HIV-1-infected patients (Everall et al., 1999; Green et al., 2019). Compared with neuronal apoptosis and HIV-encephalitis, the dendritic injury due to HIV-1 infection is more closely related to cognitive impairments among HIV-associated neurocognitive disorder (HAND) patients (Everall et al., 1999). Guha et al. (2018) compared the expression of Ng in the frontal cortex (FC) between HIV-1-positive subjects with and without HAND and the controls (Guha et al., 2018). The study found that the expression levels of Ng are reduced significantly in FC of HAND-positive patients in contrast with the uninfected individuals. Yet, a recent cross-sectional study showed that CSF Ng concentrations are in the same range for all the groups of HIV-infected patients and uninfected controls (Yilmaz et al., 2019).

#### Cerebrospinal Fluid Neurogranin in Neurosyphilis

NS, the clinical outcomes of nervous system infection of *Treponema pallidum*, can occur at any stage of syphilis (Ropper, 2019). NS is very insidious in the early stage, while its clinical manifestation in the late stage is very serious, which includes the general paresis and tabes dorsalis. Thus the early diagnosis and differential diagnosis are critical.

Intriguingly, patients with NS at a later stage general paresis of the insane (GPI) are found to have the brain pathology features of AD (Zhang et al., 2020). In a recent study, the levels of Ng and amyloid precursor protein cleaving enzyme (BACE1) in CSF and plasma, together with Aβ40, Aβ42, and t-tau in the CSF of AD patients (*n* = 23), GPI patients (*n* = 55), and NS patients (*n* = 13) were tested (Zhang et al., 2020). It found that the CSF concentrations of Ng, BACE1, and tau and the plasma BACE1 levels significantly differ among all the groups (Zhang et al., 2020). Pooling data from GPI and NS patients, both CSF tau and plasma Ng levels, are associated with cognitive scale score. These findings indicate the potential of diagnosis, differential diagnosis, and assessment of the severity of NS (Zhang et al., 2020). However, there are a few other reports, and further research is needed.

### Cerebrospinal Fluid Neurogranin in Mental Disorders

Millions of people experience mental disorders, such as schizophrenia and depression. These mental diseases are characterized by a combination of abnormal thoughts, emotions, behaviors, and perceptions (Quintero et al., 2019). Given the multifactorial complexity of these disorders, the biomarkers are supposed to assist in the early diagnosis, monitoring, and treatment selection. As a severe and complex mental disorder, schizophrenia has a lifetime prevalence of ∼1%, constituting ∼1% of the global burden of the disease (Lora et al., 2012). A genome-wide association study identified a relationship between schizophrenia and the single nucleotide polymorphism of rs12807809 in the NRGN (Stefansson et al., 2009). In recent years, several reports attempted to reveal the association between rs12807809 polymorphism and schizophrenia among different populations, but the results were controversial (Li et al., 2010; Sudesh et al., 2017). A recent meta-analysis aiming to integrate the present studies on the 12807809 polymorphism showed a statistically significant association between schizophrenia and rs12807809 polymorphism in the overall population in the allelic model (odds ratio = 1.10, 95% confidence interval 1.04–1.17). Nevertheless, the subgroup analysis revealed that a similar association only exists in Caucasians but not in Asians (Jin et al., 2019). A study of postmortem brain tissues showed a significant decrease in Ng immunostaining in both areas 9 and 32 of the prefrontal cortex (PFC) in schizophrenia compared with controls (Broadbelt et al., 2006).

In an animal study, transgenic mice overexpressing Ng in the PFC show the enhanced local plasticity and increased rate of extinction learning among different behavioral tasks, suggesting that Ng signaling in the PFC may be a specific therapeutic target for the treatment of disorders that are characterized by impaired extinction of fearful stimuli, e.g., post-traumatic stress disorder, or of reward-associated stimuli, e.g., drug addiction (Zhong et al., 2015). Electroconvulsive therapy (ECT) is a wildly used treatment for severe depression, which is considered to facilitate the neurogenesis and neural plasticity (Rotheneichner et al., 2014). A study investigated the changes of CSF Ng in response to ECT treatment in patients with depression and found that the mean CSF Ng levels do not alter within a course of ECT, but the low baseline Ng levels in the patients with major depression are positively associated with the degree of therapeutic response (Kranaster et al., 2017).

Further, a study examined CSF Ng in patients with first-episode psychosis (FEP) and HCs. It showed that CSF Ng is lower in FEP patients compared with the controls, although it is not statistically significant. In the FEP group, the significant effects of antipsychotic treatment, which is correlated to the lower levels of CSF Ng, suggest that CSF Ng is probably changed as a consequence of minimal exposure to the antipsychotic treatment (Santillo et al., 2019).

In fact, studies on the association between Ng genotypes and psychiatric disorders, particularly schizophrenia, have been carried out for many years. However, the research on the relationship between body fluids-based Ng and mental disorders is still at an early stage and is worth further exploration.

### Serum Neurogranin in Traumatic Brain Injury

Traumatic brain injury is a significant medical problem worldwide, which may cause short- or long-term synaptic changes in the CNS, resulting in an increased risk for cognitive impairment later in life (Svirsky et al., 2020). Animal studies showed that TBI could cause significant changes in axonal structure, synaptic structure, dendritic morphology, and spine density as a result of diffuse axonal injury and synaptic loss (Gao et al., 2011; Park and Biederer, 2013). A study developed a sensitive Ng sandwich ELISA to measure Ng quantitatively in serum samples from both cohorts of acute TBI patients and non-TBI controls. It found that serum Ng levels in acute TBI patients are significantly higher than those in non-TBI controls, with a ROC of 0.72 for diagnosing TBI (Yang et al., 2015). An observational emergency department study of head-injured and control patients also reached the consistent conclusions that Ng is elevated within 2–6 h after injury (Peacock et al., 2017). A recent study aimed to explore the effect of TBI on Ng by detecting the protein expression at different time points after injury (Svirsky et al., 2020). Adult male rats were subjected to either CCI group or sham group, and the expression of Ng and postsynaptic density (PSD) 95 was measured by Western blotting in the cortex and hippocampus at 1, 7, 14, and 28 days after injury. It found that the contralateral and ipsilateral hippocampus have a significant reduction in Ng levels at 1 day after CCI injury. Besides, the levels of Ng in the ipsilateral hippocampus are still significantly decreased at 7 and 14 days after CCI injury, whereas they recover to sham levels by 28 days. These results indicated that CCI lowers Ng expression in a temporal and regional specificity manner (Svirsky et al., 2020).

As a disease entity, TBI is also a risk factor for a variety of neurological diseases. Current studies suggest that Ng has potential in TBI diagnosis and disease progression. However, the lack of clinical research has limited the further transformation and application of Ng.

### Cerebrospinal Fluid and Plasma Neurogranin in Acute Ischemic Stroke

Globally, stroke (including ischemic stroke and hemorrhagic stroke) affects around 13.7 million individuals per year and is the second leading cause of death (Lindsay et al., 2019). Ischemic stroke caused by arterial occlusion is responsible for the majority of stroke cases (Campbell et al., 2019). After a stroke, a period of plasticity involving the neuronal genesis and synaptic modulation is essential to spontaneous recovery, which contains compensatory adaptation and real neurologic recovery (Felling and Song, 2015). A prospective study exploring Ng in paired CSF/plasma samples of AIS patients used both ELISA and single-molecule array (Simoa) technology for Ng measurement (De Vos et al., 2017). It showed that plasma Ng levels are only associated with the volume of cerebral infarction. Likewise, the levels of CSF Ng are significantly higher in patients with an infarction volume >5 ml than those in patients with smaller infarction volume. However, neither the symptoms severity nor long-term outcomes are correlated with Ng in plasma or CSF (De Vos et al., 2017).

In addition to AD, AIS is the only disease that has been studied to observe levels of CSF and blood Ng and their correlation. The discussed findings suggest the potential of blood Ng in reflecting brain tissue damage. However, whether the blood and CSF Ng have certain consistency is still an unavoidable issue for blood-based Ng. Recent studies have found that some kind of enzymes have the capacity of cleaving Ng and yielding specific fragments (Becker et al., 2018), which could influence the accuracy of the current detection methods. Therefore, the development of novel detection approaches is an urgent part of Ng clinical research.

## Discussion

Considerable evidence proves that a synaptic dysfunction is an early event in the pathogenesis of many neurodegenerative diseases, particularly in the AD (DeKosky and Scheff, 1990; Masliah, 2001) and PD (Jellinger, 2012). As a postsynaptic protein, Ng has been recommended as a promising biomarker for synapse loss or dysfunction (Brinkmalm et al., 2019). A series of clinical studies have confirmed the rationality, validity, sensitivity, and specificity of CSF Ng in the diagnosis for both AD dementia and prodromal AD. Thus, CSF Ng has been included in the A/T/(N) research framework of the biological definition of AD as an essential indicator of neurodegeneration (Jack et al., 2018).

However, there are still many problems in the development of the application of Ng from bench to bedside. So far, no clear evidence proves that Ng is a disease-specific biomarker, as the change of function and structure of synapse is common in the pathogenesis of different kinds of CNS diseases, indicating that the current research reports are insufficient to uncover the profile and potential application values of Ng in clinical practice. Besides, the consistency of the present results about Ng and AD is acceptable, but the other findings need more interpretation, such as the association of Ng with PD (Selnes et al., 2017; Hall et al., 2020), neuro-HIV (Guha et al., 2018; Yilmaz et al., 2019), FTD (Byrne et al., 2018; Clarke et al., 2019), etc., which in part attributes to the current measuring methods lacking high accuracy in detecting the proteins of extremely low levels both in CSF and blood.

Given that the blood samples are more accessible than CSF samples, striving has been made for replacing CSF-based biomarkers with blood-based biomarkers, especially for the diagnosis of NDs such as AD. For instance, the blood and CSF levels of neurofilament light (a promising biomarker for AD diagnosis) has been proved consistent (Fortea et al., 2018; Jack et al., 2018), indicating that it is potential to develop a blood-based, rapid, simple, portable, and easily accessible testing method for the AD screening in community populations. Frustratingly, only scattered studies have investigated blood Ng levels but failed to show a significant difference between AD patients and HCs. Also, no significant correlation between CSF and blood Ng was reported previously (De Vos et al., 2015). Besides, blood Ng has also been poorly studied in other neurodegenerative diseases. Given that the concentration of CNS biomarkers outside of the CNS is often extremely low, it is difficult to be conducted using conventional clinical assays.

Other important factors complicating the analysis include peripheral expression of Ng, the endogenous antibodies interfering with the measured results, and the proteases influence the catabolism of Ng (Zetterberg and Burnham, 2019). Since the discovery of Ng in 1990 (Watson et al., 1990), there are, to our knowledge, very few studies investigating its metabolic profile. Mass spectrometry analyses suggested that Ng is catabolized into several short C-terminal peptides, which can be identified in CSF, and only minute amounts of full-length Ng is present in CSF. Furthermore, it showed that Ng in human plasma exists as several endogenous peptides via analyzing paired plasma and CSF samples from patients with AD and HCs. Among the endogenous Ng peptides detected, CSF Ng 48–76 shows the most pronounced increase in patients with AD compared with the controls. Importantly, Ng 48–76 is also proved to be dominant in the brain tissues of AD patients. However, Ng 48–76 is not detected in plasma. These findings indicate that this particular peptide is probably brain-specific (Kvartsberg et al., 2015b). Conversely, four of the Ng peptides found only in plasma are not generated after incubation of full-length Ng in Ng-depleted plasma, indicating that some certain enzymes existing in plasma have the capacity of cleaving Ng at different sites (Kvartsberg et al., 2015b).

On the basis of current studies, Ng is expressed in the lung, spleen, bone marrow, and platelets, which may contribute to its high concentrations in blood. Due to the high plasma concentrations of Ng in normal individuals (Kvartsberg et al., 2015b), the subtle alteration is probably not detected in blood in case of chronic progressive neurodegeneration like AD. Besides, Ng is catabolized into several short C-terminal peptides; the levels of which vary in CSF and plasma, implying that the development of monoclonal anti-Ng antibodies-based testing methods is relatively difficult.

In summary, blood-based biomarkers are an important development direction in the diagnosis of neurological and mental diseases due to their many advantages compared with CSF based biomarkers. Currently, the transformation process of Ng from bench to bed has been developing rapidly in the NDs, especially in AD. In other kinds of diseases, such as PD and schizophrenia, it also has a very great potential value of transformation and application. As an essential synaptic component, Ng is a potential and promising biomarker to improve the diagnosis, prognosis and severity evaluation of the neurological and mental diseases in the future with the development of detection approaches and sample processing.

## Author Contributions

All authors listed have made a substantial, direct and intellectual contribution to the work, and approved it for publication.

## Conflict of Interest

The authors declare that the research was conducted in the absence of any commercial or financial relationships that could be construed as a potential conflict of interest.

## Abbreviations

AD: Alzheimer’s disease
AIS: acute ischemic stroke
A β: amyloid β
BACE1: precursor protein cleaving enzyme
CCI: controlled cortical impact
CJD: Creutzfeldt–Jakob disease
CNS: central nervous system
CSF: cerebrospinal fluid
DLB: dementia with Lewy bodies
FTD: frontotemporal dementia
GPI: general paresis of the insane
HD: Huntington disease
MCI: mild cognitive impairment
NDEs: neuronal-derived exosomes
NDs: neurodegenerative disorders
neuro-HIV: neuro-human immunodeficiency virus
NfL: neurofilament light
Ng: neurogranin
NRGN: neurogranin gene
NS: neurosyphilis
PD: Parkinson disease
PDD: PD with dementia
p-tau: phosphorylated tau
ROC: receiver operating characteristic
TBI: traumatic brain injury
t-tau: total tau.
